# Use of Medical-Grade Honey to Treat Clinically Infected Heel Pressure Ulcers in High-Risk Patients: A Prospective Case Series

**DOI:** 10.3390/antibiotics12030605

**Published:** 2023-03-17

**Authors:** Georgios E. Papanikolaou, Georgios Gousios, Niels A. J. Cremers

**Affiliations:** 1 GP Plastic Surgery Private Practice, P. Dagkli 1, 45444 Ioannina, Greece; 2 PharmaLife, I. Vilara 40, 45444 Ioannina, Greece; 3 Department of Gynecology and Obstetrics, Maastricht University Medical Centre, P. Debyelaan 25, 6229 HX Maastricht, The Netherlands; 4 Triticum Exploitatie BV, Sleperweg 44, 6222 NK Maastricht, The Netherlands

**Keywords:** medical-grade honey, heel-pressure ulcers, infection, antibiotic resistance, wounds, wound healing

## Abstract

Management of locally infected heel-pressure ulcers (HPUs) remains challenging, and given the increasing occurrence of infections resistant to antibiotic therapy and patients’ unwillingness to surgery, innovative and effective approaches must be considered. Medical-grade honey (MGH) could be an alternative therapeutic approach due to its broad-spectrum antimicrobial activity and healing properties. This study aimed to present the high effectiveness and safety of MGH for the conservative treatment of clinically infected HPUs. In this case series, we have prospectively studied nine patients with local signs of infected HPUs. In all cases, HPUs persisted for more than 4 weeks, and previous treatments with topical antibiotics or antiseptic products were ineffective. All patients were at high-risk to develop HPU infection due to their advanced age (median age of 86 years), several comorbidities, and permanent immobility. All wounds were treated with MGH products (L-Mesitran), leading to infection resolution within 3–4 weeks and complete wound healing without complication. Considering the failure of previous treatments and the chronic nature of the wounds, MGH was an effective treatment. MGH-based products are clinically and cost-effective for treating hard-to-heal pressure ulcers such as HPUs. Thus, MGH can be recommended as an alternative or complementary therapy in wound healing.

## 1. Introduction

Pressure ulcers (PUs) are localized damage to the skin and/or underlying soft tissues caused by pressure or shear, usually over a bony prominence [1,2]. Heel-pressure ulcers (HPUs) are the second most common type of PUs after the sacrum and the site where the most critical and severe PUs tend to develop [3,4,5]. The heel is particularly vulnerable to pressure injury due to its thin skin, and lack of fat tissue and muscle for protection and cushioning. Moreover, the limited weight-bearing area of the posterior part of the heel must sustain high-pressure forces that are exerted directly over the calcaneus.

Currently, the median incidence rate of HPUs in hospitals is estimated as 17.4% and the median prevalence rate as 11.7% [6]. The vast majority of HPUs remain superficial, involving only the skin (stage I and II) or the underlying subcutaneous tissue (stage III); and about 11% to 18% of all HPUs involve deeper tissues, such as muscle, tendon, or bone (stage IV) [6,7,8].

Particularly burdensome for the public health systems as well as for patient’s quality of life is the management of hard-to-heal HPUs, defined as an injury of the skin that persists for at least 4–6 weeks, which shows no tendency to heal despite the use of different treatment protocols [9,10]. Additional aggravating factors can be the presence of different comorbidities, especially in the elderly population, such as diabetes and peripheral arterial disease, previous surgical procedures, impaired nutritional status, and mobility problems [11,12,13]. Therefore, these patients are at a high risk to develop pressure ulcers complicated by local infection and consequently, a delay in the wound-healing process.

Clinical assessment of the wound characteristics is an important step in the selection of the appropriate treatment. Chronic ulcers can be complicated with inflammation and, therefore, microbial colonization; in addition, the early recognition of local signs and symptoms of infection is mandatory for a successful healing trajectory. A superficial increased bacterial burden is mainly characterized by wound-healing delay, moderate exudate levels, presence of debris (yellow or black necrotic tissue), and unpleasant odor from the wound [14]. A deep infection is usually presented with large ulcer dimensions, locally increased temperature, pain, edema, malodor, high exudate levels, and often bone exposure [14]. Considering the increased occurrence of infections resistant to antibiotics, new and more efficient therapies are required to effectively treat locally infected HPUs.

Honey has been used for wound healing and local infections since ancient times [15]. Medical-grade honey (MGH) is carefully selected, clean of pollutants, follows specific physicochemical characteristics, and is gamma-sterilized to guarantee its safe use for medical applications [16]. MGH has broad-spectrum antimicrobial properties principally due to its high sugar content, which creates an osmotic gradient leading to microbial dehydration and growth inhibition [17,18,19,20]. Other antimicrobial mechanisms of MGH are the acid pH, the production of low levels of hydrogen peroxide, and the release of components, such as flavonoids, methylglyoxal, and bee defensin-1, which are factors that are toxic to almost all microorganisms, but not to the healthy surrounding tissue [17,18,19,20].

Another important property of the MGH is its wound-healing activity. MGH allows for effective autolytic wound debridement, leading to the removal of necrotic tissue [21,22,23]. In addition, MGH has anti-inflammatory and antioxidative activity, creates a moist environment, and enhances the regenerative process in the wound by stimulating the formation of healthy granulation tissue and neo-epithelization [24,25,26]. MGH speeds up healing in different types of acute and chronic wounds, including pressure ulcers [27,28].

In this prospective case series, we present our experience in the treatment of clinically infected HPUs with MGH (L-Mesitran, Triticum Exploitatie BV, the Netherlands). The aim of this study is to demonstrate the effective and safe use of MGH in high-risk elderly patients with multiple comorbidities.

## 2. Results

### 2.1. Case 1

**Figure 1:**
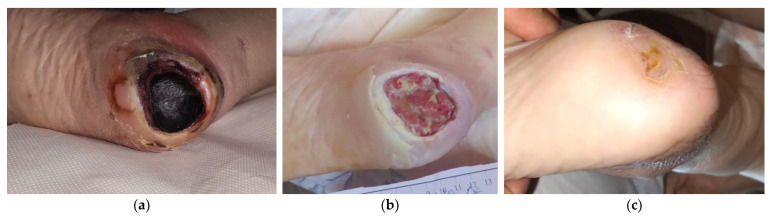
Case 1: (**a**) clinical findings at the initial examination, day 0 (start of MGH treatment); (**b**) effective debridement and healthy granulation tissue after four weeks of MGH therapy; (**c**) complete wound healing after 17 weeks of MGH therapy.

An 85-year-old female patient presented with a stage III HPU at her right foot due to prolonged immobility after hip arthroplasty (Figure 1a). Medical comorbidities included dementia, hyperlipidemia, hypertensive heart disease, and deficiency of vitamin B12 and calcium. The wound had been present for >2 months and unsuccessfully treated with neomycin sulfate topical spray. On initial evaluation, the ulcer dimensions were 5 cm in length and 5 cm in width. Local clinical signs of infection included hard necrotic eschar, low levels of exudate, pain, and delayed healing. Surgical (scalpel) debridement at the bedside was performed to remove the thick eschar. L-Mesitran^®^ Soft wound gel (MGH) was applied directly to the wound, followed by L-Mesitran^®^ Tulle (MGH) to ensure contact with the wound bed. Then, a secondary foam dressing was applied to absorb the secretions and offload the heel region. Wound-dressing changes were performed by the healthcare professional at the patient’s home at 48 h intervals. After 4 weeks, pain and exudate were considerably reduced, and healthy granulation tissue was evident (Figure 1b). Due to improved wound healing, dressing changes were transitioned to every 4 days. The HPU was completely healed after 17 weeks of MHG treatment without complications (Figure 1c).

### 2.2. Case 2

**Figure 2:**
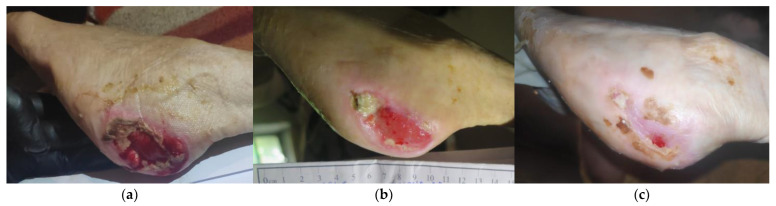
Case 2: (**a**) clinical findings at the initial examination, day 0 (start of MGH treatment); (**b**) reduction of the wound size with marginal re-epithelialization and granulation tissue formation after three weeks of MGH therapy; (**c**) complete wound healing after 12 weeks of MGH therapy.

An 88-year-old female patient presented with a stage III HPU at her left foot due to permanent immobility (Figure 2a). Relevant comorbidities included dementia, cerebrovascular disease, arterial hypertension, anemia, iron deficiency, and nephritis. The patient’s familiar ambient was non-compliant and severe malnutrition was noticed. Concomitant pressure ulcers were presented at the sacral–coccyx area and the tibial area bilaterally. The HPU was previously treated for 4 weeks with a povidone–iodine solution and a mupirocin-based topical cream, without clinical improvement. Upon presentation, the wound dimensions were 5 cm in length and 4 cm in width. Clinical signs of infections were the presence of a moderate amount of exudate, local hyperthermy, malodor, slough, and pain. Moreover, the wound edges were macerated and indented with a significant delay in the healing process. Local treatment was initiated with L-Mesitran^®^ Soft wound gel (MGH), followed by L-Mesitran^®^ Tulle (MGH). Then, a secondary foam dressing was applied to absorb the secretions and offload the heel region. Wound-dressing changes occurred at the patient’s home every 48 h by the healthcare professional. After 3 weeks of MGH treatment, the wound size reduced, granulation tissue was visible, the wound edges showed a normal re-epithelialization process, and clinical signs of infection disappeared (Figure 2b). Consequently, ulcer changes were extended to every 4 days. The HPU was completely healed after 12 weeks of MGH therapy (Figure 2c).

### 2.3. Case 3

**Figure 3:**
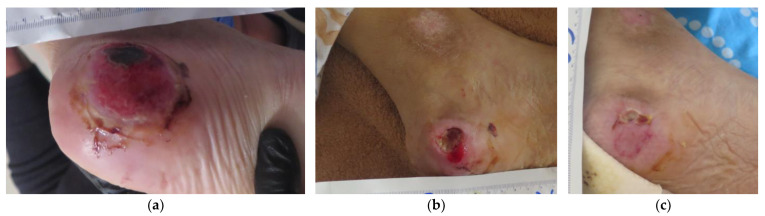
Case 3: (**a**) clinical findings at the initial examination, day 0 (start of MGH treatment); (**b**) progression of wound healing after three weeks of MGH therapy; (**c**) complete wound healing after 8 weeks of MGH therapy.

A 72-year-old female patient presented with a stage III HPU at her left foot due to permanent immobility associated with several comorbidities, including dementia, cerebrovascular disease, atrial fibrillation, myocardial infarction, arterial hypertension, and osteoporosis (Figure 3a). Previously, the wound was ineffectively cleansed for one month with soap and normal saline. On the initial presentation, her wound measured 5 cm in length and 5 cm in width. Local clinical signs of infection included erythema, low amount of exudate, debris, and delayed healing. Local treatment was initiated with L-Mesitran^®^ Soft wound gel (MGH), followed by L-Mesitran^®^ Tulle (MGH). Then, a secondary foam dressing was applied to absorb the secretions and offload the heel region. Wound-dressing changes were performed by the patient at home every 48 h intervals. At her 3-week follow-up, necrotic tissue was eliminated due to the osmotic property of the MGH products, the wound defect reduced considerably in size, and erythema disappeared (Figure 3b). Treatment was continued as per above, and complete healing was uneventfully achieved after 8 weeks (Figure 3c).

### 2.4. Case 4

**Figure 4:**
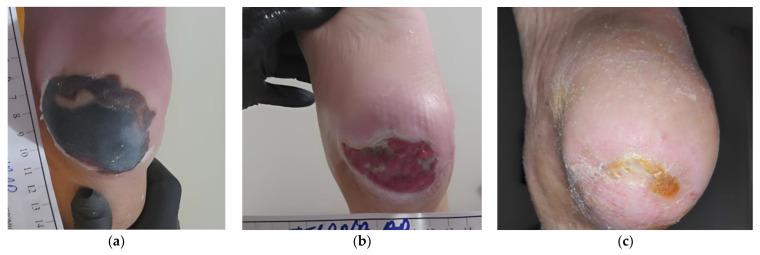
Case 4: (**a**) clinical findings at the initial examination, day 0 (start of MGH treatment); (**b**) elimination of local signs of infection and advanced wound healing after 8 weeks of MGH therapy; (**c**) complete wound healing after 24 weeks of MGH therapy.

An 86-year-old female patient presented with a stage III HPU at her left foot due to prolonged immobility after being operated on for a hip fracture (Figure 4a). Medical comorbidities included arterial hypertension, cerebrovascular disease, epilepsy, anxiety, and deficiency of vitamin D. During her hospitalization, the HPU was treated for 10 days with different wound care products, such as silver and foam dressings, all without success. Three weeks after her dismission from the hospital, the wound dimensions were 6 cm in length and 5 cm in width. Local signs of infection included the presence of a thick and large necrotic eschar, a low amount of exudate, an unpleasant odor, pain, and delayed healing. Partial surgical (scalpel) debridement was performed at the bedside and was limited secondary to pain and anticoagulation therapy. Local treatment with L-Mesitran^®^ Soft wound gel (MGH), followed by L-Mesitran^®^ Tulle (MGH), and a foam dressing was initiated to promote autolytic debridement and eliminate the remaining necrotic tissue and resolve the underlying infection. Initially, dressing changes were performed by the healthcare professional at the patient’s home daily, due to the high amount of exudate that was secreted, and to control any potential hemorrhagic diathesis. Within 1 week after MGH therapy was started, the malodor disappeared, while the autolytic debridement process was evident. Given the prosperous wound-healing progress, dressing changes interval were extended to every 4 days. After 4 weeks, the wound bed was completely clean, the defect started to be covered with new granulation and epithelial tissue, and the infection resolved (Figure 4b). The patient’s HPU was completely healed after 24 weeks of MGH treatment (Figure 4c).

### 2.5. Case 5

**Figure 5:**
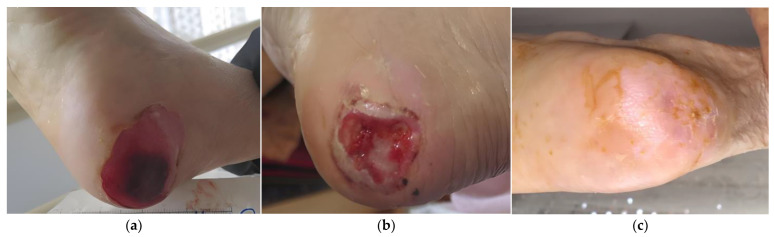
Case 5: (**a**) clinical findings at the initial examination, day 0 (start of MGH treatment); (**b**) resolution of local infection and improved wound healing after two weeks of MGH therapy; (**c**) complete wound healing after 31 weeks of MGH therapy.

A 92-year-old female patient presented with a stage III HPU at her left foot due to permanent immobility (Figure 5a). Medical comorbidities included cerebrovascular disease, rheumatic polymyalgia, and glaucoma. The wound was previously treated with povidone–iodine for one month, without any improvement. On initial observation, the HPU sized 6 cm in length and 4 cm in width. Local signs of infection included a central necrotic area with peripheric erythema, pain, and delayed healing. Treatment with L-Mesitran^®^ Soft wound gel (MGH), followed by L-Mesitran^®^ Tulle (MGH), and a foam dressing was commenced; and dressing changes were performed at the patient’s home by the health-care professional every 48 h. During the next 2 weeks, local signs of infection gradually disappeared and the wound area was reduced, and replaced by granulation and epithelial tissue (Figure 5b). Due to the positive therapeutic response, dressing changes were prolonged to every 4 days. Consequently, the patient was temporarily lost from the follow-up probably because she was non-compliant with the proposed MGH therapy. After 3 months, she showed up and the HPU was stable, but not yet healed. MGH therapy restarted as per above, and the HPU was completely healed after 31 weeks of MGH treatment without complications (Figure 5c).

### 2.6. Case 6

**Figure 6:**
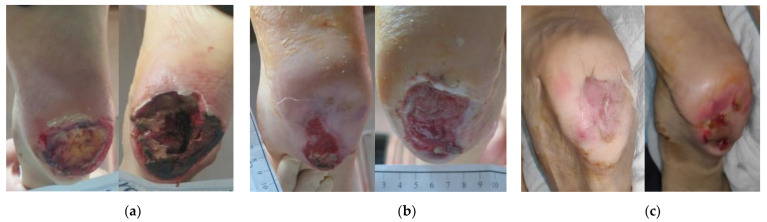
Case 6: (**a**) clinical findings at the initial examination, day 0 (start of MGH treatment); (**b**) MGH and serial surgical debridements lead to cleansing of the wound bed and progression of the wound healing after 8 weeks of treatment; (**c**) complete wound healing after 22 weeks of MGH therapy.

A 94-year-old male patient presented with a bilateral stage III HPU (Figure 6a). HPUs were caused by permanent immobility and aggravated by a prolonged hospitalization (about one month) related to uncontrolled diabetes. The patient had several comorbidities, including diabetes, cerebrovascular disease, depression, prostatic hypertrophy, anemia, and deficiency of vitamin B12. Previous treatments were not reported by his relatives. An initial examination was held 10 days after his dismission from the hospital, where the right HPU measured 6 cm in length and 5 cm in width; and his left HPU measured 7 cm in length and 7 cm in width. Local infection was evident by the presence of large heels defect, extended necrosis, heavy exudate, malodor, erythema, pain, and delayed healing. The initial therapeutic protocol included serial surgical (scalpel) debridement at the bedside with a 2-week interval in between, followed by the application of L-Mesitran^®^ Soft wound gel (MGH), L-Mesitran^®^ Tulle (MGH), and a foam dressing. MGH dressing changes were performed at the patient’s home daily by his relatives to permit effective drainage of the exudate and achieve an osmotic cleansing of the wound bed from the extended necrotic tissue. After 8 weeks of combined treatment, clinical signs of infection were resolved, the wound bed was noticeably cleansed, and new granulation tissue started to fill the heel defect (Figure 6b). Since the healing process progressed successfully, the dressing was changed every 3 days. Finally, complete HPU healing was achieved after 22 weeks of MGH treatment without complications (Figure 6c).

### 2.7. Case 7

**Figure 7:**
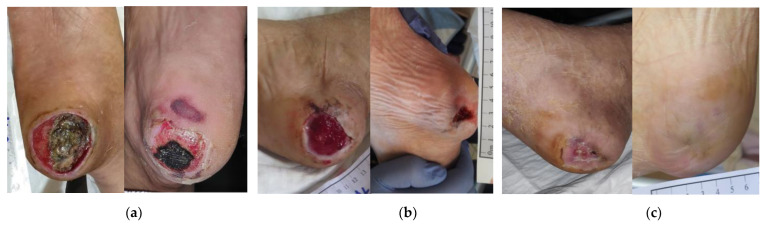
Case 7: (**a**) clinical findings at the initial examination, day 0 (start of MGH treatment); (**b**) MGH effectively induced autolytic debridement, resolution of the local signs of infection, and advanced wound healing after 2 weeks of treatment; (**c**) complete wound healing after 108 days of MGH therapy for the right heel and 17 weeks of MGH therapy for the left heel.

A 59-year-old female patient presented with a stage III bilateral HPU (Figure 7a). She was hosted in a boarding house for chronically mentally ill patients. She suffered from severe psychotic disorders, which combined with a lack of compliance and prolonged immobility led to the development of the HPUs. Previously, the wounds were ineffectively treated for one month with a povidone-iodine solution. On initial examination, the right HPU measured 6 cm in length and 5 cm in width; and his left HPU measured 5 cm in length and 4 cm in width. Local signs of infection included a high amount of exudate, malodor, necrosis, slough, and delayed healing. The level of pain was impossible to be assessed due to her psychiatric conditions. Surgical (scalpel) debridement was impossible due to the lack of patient compliance, and the commencement of local therapy with L-Mesitran^®^ Soft wound gel (MGH), L-Mesitran^®^ Tulle (MGH), and a foam dressing were decided. Initially, dressing changes were performed by the healthcare professional every 48 h. After 2 weeks, the autolytic effect of MGH was evident, and signs of infection resolved (Figure 7b). Moreover, the wound area was reduced and gradually replaced by healthy granulation and epithelial tissue. Since the wounds became more superficial and the amount of exudate was considerably reduced, the interval of dressing changes was prolonged to every 4 days. The right HPU was completely healed after 108 days of MGH treatment, while the left HPU healed after 17 weeks of MGH treatment (Figure 7c).

### 2.8. Case 8

**Figure 8:**
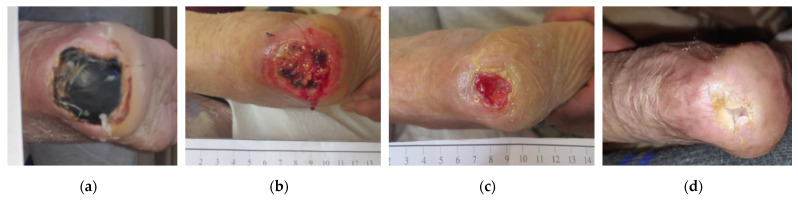
Case 8: (**a**) clinical findings at initial examination, day 0 (start of MGH treatment); (**b**) the MGH products allowed the softening of the eschar and permitted an easy surgical removal of the necrotic tissue with concomitant advanced wound healing after 4 weeks of treatment; (**c**) elimination of local signs of infection and further wound healing progression after 8 weeks of MGH therapy; (**d**) complete wound healing after 13 weeks of MGH therapy.

An 87-year-old female patient presented with a stage III HPU on her right foot due to prolonged immobility after being operated on for a hip fracture (Figure 8a). Medical history included arterial hypertension, organic psychotic disorder, and an already established permanent immobility. The HPU was unsuccessfully treated for 1.5 months with different wound care products, such as silicone foam dressings and neomycin sulfate topical spray. On initial evaluation, the wound dimensions were 4 cm in length and 4 cm in width. Local signs of infection included the presence of a thick necrotic eschar, erythema, pain, malodor, and delayed healing. Bedside conservative debridement was impossible due to the patient’s intolerance associated with her psychotic disease and it was decided to start the therapy with L-Mesitran^®^ Soft wound gel (MGH), L-Mesitran^®^ Tulle (MGH), and a foam dressing. Wound-dressing changes were performed by the healthcare professional at the patient’s home at 48 h intervals. Within 4 weeks, the osmotic property and moist environment provided by the MGH products allowed the softening of the eschar and permitted an easy surgical removal of the necrotic tissue (Figure 8b). Moreover, all signs of local infection disappeared, wound size decreased, and new granulation tissue was evident at the wound bed. During the next 4 weeks, the wound healing further progressed, and dressing changes were transitioned to every 4 days (Figure 8c). The HPU healed after 13 weeks of MGH treatment (Figure 8d).

### 2.9. Case 9

**Figure 9:**
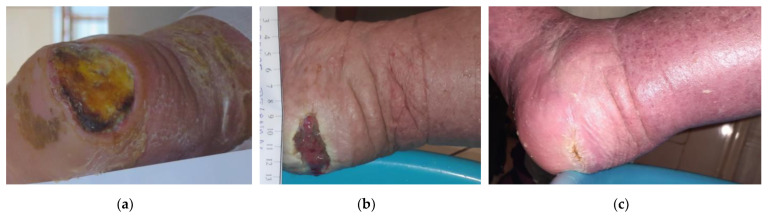
Case 9: (**a**) clinical findings at initial examination, day 0 (start of MGH treatment); (**b**) MGH products induced autolytic debridement and promoted wound healing after 4 weeks of treatment; (**c**) complete wound healing after 20 weeks of MGH therapy.

A 78-year-old female patient presented with a stage III HPU at her right foot caused by permanent immobility (Figure 9a). Relevant comorbidities included peripheral arterial disease, arterial hypertension, Parkinson’s disease, and osteoporosis. The HPU was ineffectively treated for 15 days with a povidone–iodine solution. She was found to have a wound sized 6 cm in length and 3 cm in width. Local clinical signs of infection included severe edema, erythema, debris, moderate level of exudate, and delayed healing. Topical treatment was initiated with L-Mesitran^®^ Soft wound gel (MGH), L-Mesitran^®^ Tulle (MGH), and a foam dressing. Dressing changes were performed by the healthcare professional at the patient’s home every 48 h. After 4 weeks, the ulcer improved with the elimination of necrotic tissue, the appearance of healthy tissue, and no evidence of signs of local infection (Figure 9b). Due to the positive healing process, the dressing changes were extended to every four days. The HPU completely healed after 20 weeks of MGH therapy without complications (Figure 9c).

## 3. Discussion

Non-healing wounds are often complicated by local contamination or infection caused by various species of microorganisms. The bacteria in the wound can be protected by a barrier of extracellular matrix forming a biofilm, which is particularly resistant to different antibiotics [29]. The increasing occurrence of infections resistant to antibiotic therapy necessitates the development of alternative and improved treatment approaches. In our case series, all HPUs were successfully treated with MGH products. Previous treatments with different antiseptic or antibiotic products were without adequate response. Local clinical signs of infection gradually decreased and completely resolved within a time range from 1–4 weeks, in concordance with other studies [30,31,32]. In this study, wound swabs and microbial culturing were not taken and performed since the diagnosis was made by clinical assessment of different signs of local infection, such as necrosis, pain, malodor, erythema, warmth, edema, exudate, and delayed healing. In addition, swabs can be costly, and not always precise due to the presence of normal cutaneous bacterial flora. Moreover, it can also be considered redundant when infections manifest only locally because MGH exerts broad-spectrum antimicrobial activity irrespective of their antibiotic resistance profile and is even effective against biofilms. However, from a scientific point of view, taking swabs must be considered in future studies, as this can help to demonstrate the clinical broad-spectrum antimicrobial efficacy of MGH.

In all presented cases, MGH products were used as monotherapy and none of the patients received systemic antibiotic therapy. Given the increased antibiotic resistance, MGH is a promising therapeutic approach to treat wound infections and enhance the healing process. We did not observe any adverse effects, and with the quick recession of local signs of infection and the positive healing response, the wound-dressing changes were extended, reducing in this way the total cost of the therapeutic protocol. Several studies proved the broad-spectrum antimicrobial activity of MGH against common wound microorganisms, including *Staphylococcus aureus* (including methicillin-resistant *Staphylococcus aureus, MRSA*), *Pseudomonas aeruginosa*, and *Escherichia coli*, even in cases where antibiotics were ineffective [18,30,31,32,33,34]. Recent reviews provide an extensive list of microorganisms with susceptibility to the antimicrobial activity of MGH [19,35]. Furthermore, the development of bacterial resistance after repeated use of MGH materials is thought to be unlikely, attributed mainly to the natural origin of the honey and its multiple antimicrobial components and mechanisms [17,36].

Our study included patients with a high risk to develop infections at their HPUs. In those patients, it is important to promptly identify and, if possible, correct any systemic or local factors that can lead to non-healing HPU, further complicated with inflammation and infection. Eight of the nine patients were elderly, with an average age of 82 years old. Age >65 years is frequently associated with impaired nutritional status and limited mobility usually in hospitalized and nursing/community care patients, leading to HPUs [11,37]. Moreover, all our patients had several comorbidities, presented mainly with dementia, cerebrovascular ischemic disease, and cardiovascular disease. Mental status emerged as a significant risk factor associated with HPU development [37,38], while cardiac and cerebral vascular disease can be associated with peripheral perfusion issues, which caused delaying wound healing [6,39,40,41]. Six patients presented with permanent immobility due to their advanced age, plegia, and mental disorders, and four of them had concomitant pressure ulcers in other body regions. Immobility is considered a crucial prognostic factor for the development of pressure ulcers, and different interventions must be taken to offload the pressure and prevent any ischemic injury [37,40]. In addition, three patients underwent orthopedic surgery, and they developed HPUs secondary to their prolonged immobility. Surgery is an important independent risk factor for the emergence of HPUs, especially in elderly hospitalized patients [11].

All patients had a stage III HPU with a mean length of 5.54 cm and width of 4.64 cm (length range 4–7 cm, width range 3–7 cm). Six patients presented with necrotic tissue, in four of which the eschar was effectively removed by MGH combined with limited surgical (scalpel) debridement at the bedside, and in the other two cases only MGH products were able to stimulate the autolytic debridement process and efficiently clean the wound bed. Large (>4 cm) and deep (stage III and IV) HPUs are usually prone to superficial bacterial contamination or deep wound infection and, thus, complicated with local inflammation, osteomyelitis, or systemic sepsis, require urgent surgical intervention to save the patient’s life [42]. Operative management of HPUs includes partial or total calcanectomy, revascularization techniques, free flaps, and amputation [43]. MGH can be an alternative therapeutic option to surgery, mainly in patients with age-related comorbidities, locally large and infected HPU, where operative intervention is contraindicated or not desired from the patients [44].

We used MGH materials, which positively affected the wound healing process, initially through autolytic debridement, resolution of clinical infection, and anti-inflammatory medication; and, in a second phase, promoting granulation tissue formation, neo-angiogenesis, and re-epithelialization. The healing time ranged from 2–7 months (mean 128 days, median 118 days), in accordance with other case series [30,31,32]. All wounds were completely healed without complications, improving the quality of life of the patients and their relatives. MGH exerts a broad-spectrum healing activity and can be effectively used to treat different types of wounds, such as diabetic foot, vascular ulcers, infected traumatic or surgical wounds, burn injuries, and neonatal/pediatric wounds [24,30,31,32,45]. In addition, MGH therapy shows much promise outside the regular scope of topical cutaneous wound care in non-conventional applications and indications [46].

## 4. Materials and Methods

### 4.1. Patients

In this prospective observational case series study, we used MGH wound care products to treat patients with non-healing HPUs. Inclusion criteria were having an HPU lasting more than 4 weeks, the presence of local signs of bacterial contamination or infection, and patient consent. Exclusion criteria were having an allergy to bee stings or MGH, systemic signs of infection or inflammation, and patient non-consent.

A total of nine patients (eight women and one man) developed HPUs, of which six patients were due to permanent immobility and three patients due to prolonged immobility post-orthopedic surgery. All patients were recruited prospectively during a 41-month period (April 2019 to August 2022), with a mean follow-up period of 114 days (range 57–218 days, median 118 days). During this period, there was no treatment failure with L-Mesitran products. Data were limited due to loss to follow-up, and death associated to advanced age and severe comorbidities. The average age was 82 years (range 59–94 years, median 86 years), and they all had several comorbidities. In all, seven patients had unilateral HPU and two patients had bilateral HPU. All HPUs were stage III and, upon presentation, the mean length was 5.54 cm (range 4–7 cm) and the mean width was 4.64 cm (3–7 cm), while five patients had concomitant pressure ulcers elsewhere in the body.

Different previous treatments, including topical antiseptic or antibiotic products, were ineffective. The diagnosis of wound infection was made through clinical assessment and based on signs and symptoms in and around the HPU. In four patients, we performed local surgical (scalpel) debridement at the bedside. All HPUs treated with MGH were completely healed without any complication within a mean time of 128 days (range 57–218 days, median 118 days).

### 4.2. L-Mesitran Wound Care Products and Therapeutic Interventions

L-Mesitran (www.mesitran.com, Triticum Exploitatie BV, Maastricht, The Netherlands) manufactures a variety of MGH-based products designed to treat different types of skin wounds such as pressure ulcers. L-Mesitran Soft (L-MS) is a hydro-active antibacterial wound gel containing 40% MGH. L-MS is applied in contact with the HPU, creating a moist wound-healing environment. This facilitates the autolysis of necrotic and devitalized material, provides bacterial growth inhibition, and promotes the wound-healing process. L-Mesitran Tulle (L-MT) is a non-adhering antibacterial dressing impregnated with L-MS gel. L-MT can be easily applied and used for infected superficial or deep wounds. Moreover, L-MT prevents the secondary dressing from adhering to the wound bed. In all presented cases, L-MS and L-MT were applied in combination and covered with a secondary foam dressing to control the exudate amount and offload the heel region. Initially, the dressing changes were performed at the patient’s home by the wound care professional or by the patient’s relatives.

Wound characteristics and photographic documentation at the initial presentation and subsequent follow-up visits were collected and reviewed to assess the wound infection response to MGH therapy and evaluate the wound-healing progress. The patient’s demographic data and treatment protocol are summarized in Table 1.

## 5. Conclusions

In the present case series, MGH-based products improved the clinical outcome of hard-to-heal HPUs in elderly patients with multiple and severe comorbidities. MGH is a safe and effective therapeutic approach for locally clinical infected HPUs, and can be proposed as an alternative or complementary to antibiotics and surgery. Furthermore, MGH-based products are easy to apply at home and are cost-effective. This will lead to improving the patient’s quality of life.

## Figures and Tables

**Table 1:** Demographic data and wound course overview of the presented cases.

Case #	Gender/Age (Years)	HPU Location/ Dimensions (cm)	HPU Etiology	Relevant Comorbidities	Previous Treatments	Local Signs of Infection	Time for Infection Resolution (Weeks)	Time for Wound Healing (Days)
1	Female 85	Right 5 × 5	Hip arthroplasty	Hypertensive heart disease, hyperlipidemia, dementia, deficiency of calcium and vitamin B12	Neomycin sulfate topical spray	Necrotic eschar, exudate, pain, delayed healing	4	117
2	Female 88	Left 5 × 4	Permanent immobility	CVD, AHT, dementia, anaemia, iron deficiency, nephritis	Povidone-iodine solution, mupirocin-based topical cream	Hyperthermia, exudate, malodour, pain, slough	3	86
3	Female 72	Left 5 × 5	Permanent immobility	CVD, AHT, dementia, myocardial infarction, atrial fibrillation, osteoporosis	Normal saline and soap	Debris, erythema, exudate, delayed healing	3	57
4	Female 86	Left 6 × 5	Hip fracture surgery	CVD, AHT, epilepsy, anxiety, deficiency of vitamin D	Silver and foam dressings	Necrotic eschar, exudate, malodor, pain, delayed healing	4	169
5	Female 92	Right 6 × 4	Permanent immobility	CVD, rheumatic polymyalgia, glaucoma	Povidone-iodine solution	Central necrotic area, peripheric erythema, pain, delayed healing	3	218
6	Male 94	Right 6 × 5, Left 7 × 7	Permanent immobility	CVD, diabetes mellitus type 2, benign prostatic hyperplasia, anaemia, deficiency of vitamin B12	No reported	Necrotic tissue, exudate, malodour, erythema, pain, delayed healing	8	155
7	Female 59	Right 6 × 5, Left 5 × 4	Permanent immobility	Psychotic disorders, urinary incontinence, lower limb oedema	Povidone–iodine solution	Necrotic tissue, exudate, malodor, delayed healing	2	108 (right) 118 (left)
8	Female 87	Right 4 × 4	Hip fracture surgery	AHT, organic psychotic disorder	Silicone foam dressings, neomycin sulfate topical spray	Necrotic eschar, erythema, malodor, pain, delayed healing	4	89
9	Female 78	Right 6 × 3	Permanent immobility	AHT, peripheral arterial disease, Parkinson’s disease, osteoporosis	Povidone–iodine solution	Debris, erythema, exudate, edema, delayed healing	4	137

Abbreviations: HPU: heel pressure ulcer, CVD: cerebrovascular disease, AHT: arterial hypertension.

## Data Availability

The data that support the findings of this study are available from the corresponding author upon reasonable request. All data relevant to the study are included in the article.
